# Expanding the limits of endoscopic intraorbital tumor resection using 3-dimensional reconstruction^☆^

**DOI:** 10.1016/j.bjorl.2017.11.010

**Published:** 2017-12-26

**Authors:** Luciano Lobato Gregorio, Nicolas Y. Busaba, Marcel M. Miyake, Suzanne K. Freitag, Benjamin S. Bleier

**Affiliations:** a Universidade Federal de São Paulo (UNIFESP), Escola Paulista de Medicina, Departamento de Otorrinolaringologia e Cirurgia de Cabeça e Pescoço, São Paulo, SP, Brazil; b Massachusetts Eye and Ear Infirmary, Department of Otolaryngology-Head and Neck Surgery, Boston, United States; c Coordenação de Aperfeiçoamento de Pessoal de Nível Superior (CAPES), Brasília, DF, Brazil; d Harvard Medical School, Department of Otology and Laryngology, Boston, United States; e Faculdade de Ciências Médicas da Santa Casa de São Paulo, Departamento de Otorrinolaringologia, São Paulo, SP, Brazil; f Harvard Medical School, Massachusetts Eye and Ear Infirmary, Ophthalmic Plastic Surgery Service, Boston, United States

**Keywords:** Endoscopic endonasal approach, Orbital tumors, Orbit, Nasal surgical procedures, Otorhinolaryngologic surgical procedures, Abordagem endonasal endoscópica, Tumores orbitais, Órbita, Procedimentos cirúrgicos nasais, Procedimentos cirúrgicos otorrinolaringológicos

## Abstract

**Introduction:**

Endoscopic orbital surgery is a nascent field and new tools are required to assist with surgical planning and to ascertain the limits of the tumor resectability.

**Objective:**

We purpose to utilize three-dimensional radiographic reconstruction to define the theoretical lateral limit of endoscopic resectability of primary orbital tumors and to apply these boundary conditions to surgical cases.

**Methods:**

A three-dimensional orbital model was rendered in 4 representative patients presenting with primary orbital tumors using OsiriX open source imaging software. A 2-Dimensional plane was propagated between the contralateral nare and a line tangential to the long axis of the optic nerve reflecting the trajectory of a trans-septal approach. Any tumor volume falling medial to the optic nerve and/or within the space inferior to this plane of resectability was considered theoretically resectable regardless of how far it extended lateral to the optic nerve as nerve retraction would be unnecessary. Actual tumor volumes were then superimposed over this plan and correlated with surgical outcomes.

**Results:**

Among the 4 lesions analyzed, two were fully medial to the optic nerve, one extended lateral to the optic nerve but remained inferior to the plane of resectability, and one extended both lateral to the optic nerve and superior to the plane of resectability. As predicted by the three-dimensional modeling, a complete resection was achieved in all lesions except one that transgressed the plane of resectability. No new diplopia or vision loss was observed in any patient.

**Conclusion:**

Three-dimensional reconstruction enhances preoperative planning for endoscopic orbital surgery. Tumors that extend lateral to the optic nerve may still be candidates for a purely endoscopic resection as long as they do not extend above the plane of resectability described herein.

## Resumo

**Introdução:**

A cirurgia orbital endoscópica é um campo emergente e são necessárias novas ferramentas para auxiliar no planejamento cirúrgico e determinar os limites da ressecabilidade tumoral.

**Objetivo:**

Usar a reconstrução radiográfica tridimensional para definir o limite lateral teórico de ressecabilidade endoscópica de tumores orbitais primários e aplicar essas condições de limites a casos cirúrgicos.

**Método:**

Um modelo orbital tridimensional foi aplicado a quatro pacientes representativos com tumores orbitais primários utilizando o *software* de imagem de fonte aberta OsiriX. Um plano bidimensional foi propagado entre a narina contralateral e uma linha tangencial ao eixo longo do nervo óptico que reflete a trajetória de uma abordagem transeptal. Qualquer volume de tumor situado medialmente ao o nervo óptico e/ou dentro do espaço inferior a esse plano de ressecabilidade foi teoricamente considerado ressecável, independentemente de quão longe ele se estendia até o nervo óptico, pois a retração do nervo seria desnecessária. Os volumes reais do tumor foram então sobrepostos sobre esse plano e correlacionados com os resultados cirúrgicos.

**Resultados:**

Entre as quatro lesões analisadas, duas eram totalmente mediais ao nervo óptico, uma se estendia lateralmente ao nervo óptico, mas permaneceu inferior ao plano de ressecabilidade e uma se estendia lateralmente ao nervo óptico e superior ao plano de ressecabilidade. Conforme previsto pelo modelo tridimensional, uma ressecção completa foi obtida em todas as lesões, exceto uma, que transgrediu o plano de ressecabilidade. Nenhuma nova diplopia ou perda de visão foi observada em qualquer paciente.

**Conclusão:**

A reconstrução tridimensional melhora o planejamento pré-operatório para a cirurgia orbital endoscópica. Os tumores que se estendem lateralmente ao nervo óptico podem ainda ser candidatos à ressecção puramente endoscópica, desde que não se estendam além do plano de ressecabilidade aqui descrito.

## Introduction

Endoscopic orbital surgery is a rapidly growing field and many studies have proven the efficacy of the exclusive endoscopic approach for management of benign and malignant intraorbital tumors.^1^, ^2^, ^3^, ^4^ As with any nascent field, new tools are required to assist with surgical planning and to ascertain the limits of tumor resectability. While algorithms have been proposed to assist in the choice of approach,^5^ these studies have relied on traditional tri-planar imaging to determine tumor morphology and lateral extension. However, the compact, conal structure of the orbital apex often obscures the precise relationship between the optic nerve and the tumor mass which, in turn, has led to conservative recommendations regarding the lateral extent of tumor resectability. Furthermore, the complex shape of the tumor as it insinuates through the apical neurovascular structures can lead to significant errors in estimation of tumor volume by tri-planar measurement. This feature becomes particularly important when trying to assess whether a lesion has been fully resected based on gross inspection of the specimen.

3-Dimensional reconstruction and analysis of planar images has become increasingly useful thanks to the proliferation of third party Digital Imaging and Communications in Medicine (DICOM) viewing software such as OsiriX (Pixmeo Geneva, Switzerland). These reconstructions are able to overcome the described limitations of planar imaging of the orbital apex as the intimate relationship between the optic nerve and the lesion can be visualized from the optic chiasm and the globe. The purpose of this study was to therefore determine whether 3-dimensional reconstruction could be used to create a precise boundary to describe the lateral limit of endoscopic tumor resectability and to accurately characterize the volume of representative orbital lesions.

## Methods

Approval of this study was obtained through the Human Studies Committee – Institutional Review Board (Protocol n° 754915-8 (15-068H)). Four patients with primary orbital tumors representative of distinct tumor epicenters who underwent endoscopic intraorbital surgery between January 2014 and May 2015 were selected. Computed tomography (CT) scans (100 kV tube voltage, 600–800 mAs intensity without modulation, and temporal resolution 125–625 ms) were acquired for each patient and imported in OsiriX Software x6.5.2 32-bit. The region-of-interest (ROI) tool was used to identify the optic nerve (ON), extraocular muscles and tumor in successive axial cuts. Three-dimensional volume rendering was used to create a reconstruction of the relationship between the bony orbit, tumor and ON and also to calculate the tumor volume.

An oblique parasagittal line was applied along the long axis of the ON dividing it into medial and lateral halves along its entire length. A 2-dimensional plane was then propagated between the contralateral nare and the line describing the long axis of the ON. This plane, termed the plane of resectability (POR), reflects the trajectory of a trans-septal approach to the orbit. According to our criteria, any tumor volume falling medial to the ON and/or inferior to the POR was considered theoretically resectable regardless of its lateral extent. This is due to the fact that the dissection can proceed inferior the ON without requiring nerve retraction. Actual tumor volumes from the representative patients were then superimposed over this plane and correlated with surgical outcomes.

## Results

**Figure 1:**
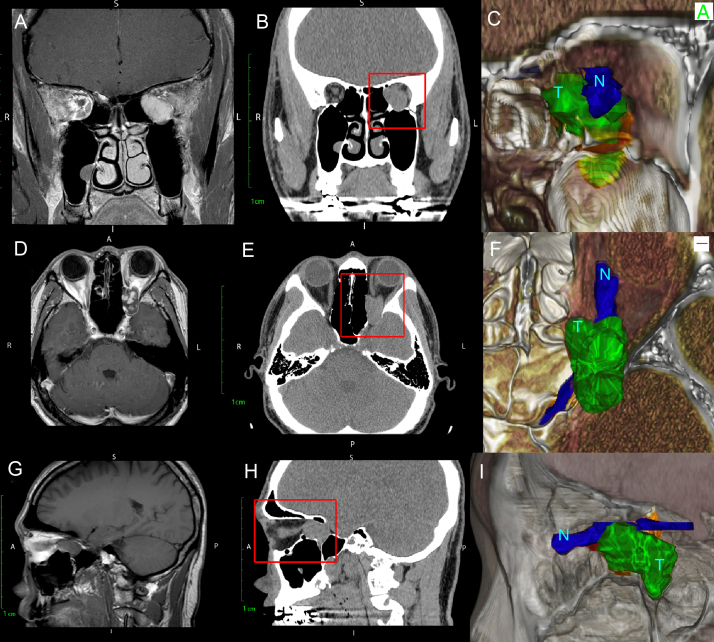
Comparison of T1-weighted MRI (A, D, G) with CT scan (B, E, H) and with 3D rendered (C, F, I) of Patient 1 with a massive orbital tumor. Note how the three-dimensional rendering gives depth to the image and improves the distinction between the ON (N) and orbital Tumor (T) which cannot be fully distinguished on either CT or MRI.

**Figure 2:**
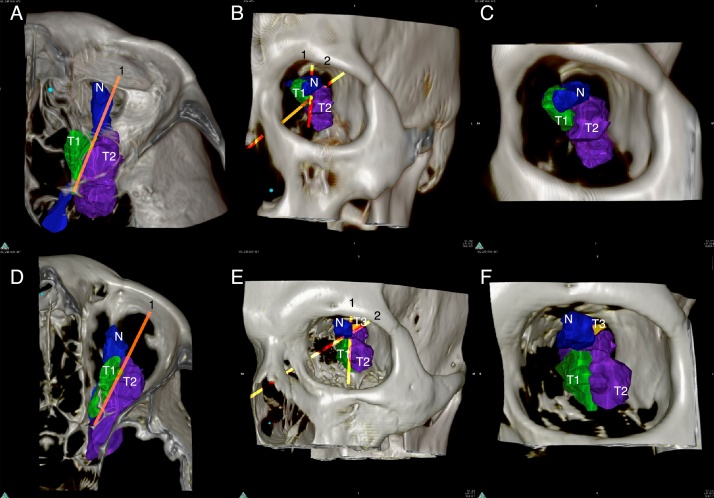
3D rendered orbital tumors of Patients 3 (A, B, C) and 4 (D, E, F). Line 1 represents the long axis of the Optic Nerve (N) while Line 2 represents the plane of resectability. Note how these lines divide the tumor into 3 zones (T1) easily resectable, (T2) resectable and (T3) unresectable.

Of the four patients studied, the relationship between the optic nerve and tumor volume could be clearly delineated following 3-dimensional reconstruction (Fig. 1). Three patients were deemed resectable according to our imaging criteria and underwent successful endoscopic gross total resection intraoperative (Table 1). Patients 2 and 3 had tumor volumes, which extended lateral to the optic nerve but remained inferior to the POR. Patient 4 was found to have a tumor volume which extended both lateral to the ON and superior to the POR (Fig. 2). This patient was deemed unresectable and underwent debulking and biopsy which was consistent with a solitary fibrous tumor.

**Table 1:** Comparison of predicted tumor volume by 3D rendering to final pathology.

Patient	Location	Pathology volume	CT volume	CT discrepancy from pathology (%)	3D volume	3D discrepancy from pathology (%)	Tumor volume lateral to the ON	Tumor volume superior to POR
1	Optic canal	0.03	0.17	496.94	0.05	151.94	0	0
2	Extraconal	3.7	7.74	206.45	4.02	107.44	0.77	0
3	Intraconal	0.39	1.43	362.63	0.48	121.69	0.1	0
4	Intraconal	–	7.79	–	4.61	–	3.9	0.17

All volumes are calculated in cm^3^.

**Figure 3:**
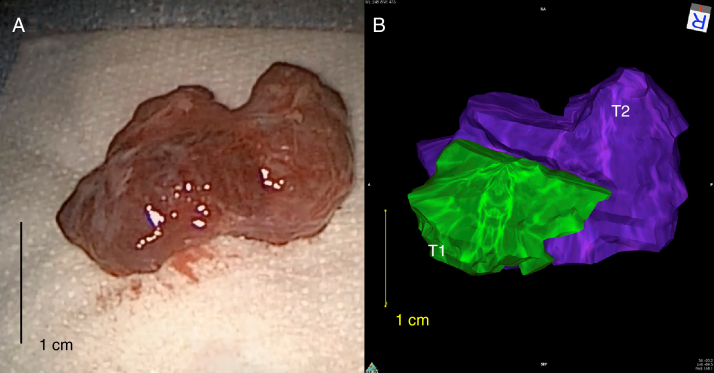
Comparison between resected orbital tumor and the 3-dimensional rendered tumor in Patient 2 demonstrating a close concordance in tumor size and morphology. Note in panel B that the different zones of the tumor were identified: (T1-green) easily resected tumor, (T2-purple) resectable tumor.

The 3-dimensional reconstruction software was significantly more accurate in predicting the gross tumor volume than traditional triplanar calculations (Fig. 3). The mean (±standard deviation) percent triplanar overestimation of the tumor volume was 355.34 ± 145.38% vs. 127.02 ± 22.72% (*p* = 0.03, Student's *t*-test) using 3-dimensional rendering.

## Discussion

The choice of surgical approach when addressing an intraorbital lesion depends on many factors including anticipated pathology, size, morphology, and location. Traditional teaching has held that endoscopic approaches to the orbit must be restricted to lesions, which remain medial to the optic nerve. As the field of endoscopic orbital surgery expands, these restrictions continue to be challenged as new diagnostic and surgical approaches are developed.^6^

Compartmentalization of the intraconal space based on its fixed neurovascular structures may help the surgeon to safely remove intraorbital lesions.^7^ However, preoperative visualization of the discreet relationship between the tumor and the optic nerve along its entire length is exceedingly difficult due to the compact neuroanatomy of the orbital apex. The advent of 3-dimensional reconstructive software enables the end user to easily import traditional triplanar imaging studies and create an accurate reconstruction of the relationship between the lesion, the ON, and any other relevant bony and muscular orbital structures.

Our findings demonstrate that these reconstructions may also be used to more precisely define the lateral limits of endoscopic approaches.^5^, ^8^ By taking into account the oblique pathway of the optic nerve and the trajectory of a transseptal approach, we have defined a novel safe plane of resection. Consequently, the criteria for endoscopic resection may be expanded to include any tumor medial to the optic nerve and/or inferior to the POR, regardless of its lateral extent.

Furthermore, the reconstructive software described herein may be used to faithfully reconstruct the morphology and volume of the orbital tumor. This feature becomes significantly important when assessing the completeness of the resection of the gross specimen. By comparing the intraoperative specimen to the preoperative reconstruction, the surgeon may more readily be able to determine whether the tumor was completely resected. This is extremely valuable in preventing the need for further surgical exploration thereby reducing the operative time, the potential for further neurovascular injury, and the requirement of intraoperative or perioperative imaging.

## Conclusion

Preoperative 3-dimensional reconstruction of orbital tumors represents a valuable diagnostic technique to evaluate the relationship between the tumor and the optic nerve as well as accurately determine the tumor volume and morphology. Using this technique, we have defined a novel plane of resectability, termed the “POR”, which challenges the conventional teaching that tumors lateral to the optic nerve should not be approached endoscopically. Based on our findings, the criteria for an endoscopic approach to the orbit can be expanded to lesions, which lie medial to the optic nerve and/or are inferior to the plane of resectability regardless of their lateral extent.

## Conflicts of interest

The authors declare no conflicts of interest.
